# Data on soil properties and halophilic bacterial densities in the Na Si Nuan Secondary Forest at Kantharawichai District, Maha Sarakham, Thailand

**DOI:** 10.1016/j.dib.2019.104582

**Published:** 2019-09-28

**Authors:** Kannika Chookietwattana, Thalisa Yuwa-amornpitak

**Affiliations:** a Department of Biotechnology, Faculty of Technology, Mahasarakham University, Maha Sarakham, Thailand

**Keywords:** Soil properties, Saline soil, Soil bacterial density, Halophilic bacteria

## Abstract

Saline soil is one of the most crucial problems of arid and semiarid regions because it reduces growth of plant and microorganisms. In Thailand, the inland saline soils are found mostly in the northeastern part especially in Maha Sarakham Province where 85% of the province has geological characteristics as rock salt beds. Saline soil often experiences low soil fertility problems which multiply the adverse effects on plant growth. Interestingly, the Na Si Nuan Secondary Forest, Kantharawichai District, Maha Sarakham, is not affected by salinity although almost the entire province of Maha Sarakham is salt-affected area. Saline soil is a habitat of halophilic bacteria. Bacteria are the most important microorganisms contribute to soil fertility and soil health. Thus data regarding the density of culturable halophilic bacteria and soil properties in this forest soil is useful for reclamation of saline soil and helping to sustain the forest ecosystem.

**Table:** Specifications Table

Subject	Environmental Sciences
Specific subject area	Environmental Microbiology and Soil Science
Type of data	Tables and figures
How data were acquired	Laboratory analysis of soil properties, quantifying halophilic bacterial densities using soil dilution technique and spread plate technique on the halobacteria medium containing different concentrations of sodium chloride
Data format	Raw and analysis
Parameters for data collection	Soil samples were collected seasonally. Within the day of sampled, they were subjected for enumeration of culturable halophilic bacteria. The remaining soil samples were analyzed for some physical and chemical properties as soon as possible.
Description of data collection	Analysis of seasonal soil physical and chemical properties (pH, soil texture class, organic matter, total nitrogen, available phosphorus and available potassium) and the density of culturable halophilic bacteria (non-, slightly-, and moderately-halophilic bacteria).
Data source location	Soil samples were collected in the Na Si Nuan Secondary Forest, Maha Sarakham, Thailand (latitude 16°20′N and longitude 103°12′E).
Data accessibility	Data incorporated within this article.

**Table:** 

**Value of the Data** • The data could help explaining the seasonal dynamics of soil chemical properties and bacterial density in a secondary forest. • The data provides baseline to study the diversity and functioning of bacteria and the soil properties. • The data may serve as benchmarks for other groups working or studying in the field of soil microbial ecology either saline soil or forest soil. • The data can be useful for policy makers and all related stakeholders working in the fields of pedology and forestry by imposing proper measures either for reclamation of saline soil or sustainable of the forest ecosystem.

## 1 Data

The data are from the 14-sampling plots investigating the seasonal dynamics of main physical and chemical soil properties and the density of non-, slightly-, and moderately halophilic bacteria. Main physical and chemical properties of soil collected in three seasons (hot, rainy and cool) from Na Si Nuan Secondary Forest, Kantharawichai District, Maha Sarakham Province, Thailand, are summarized in Table 1, Table 2. The status of soil properties were assessed against the criteria from Land Classification Division and FAO Project Staff [1], Landon [2], and Soil Survey Division Staff [3] (Table 3).

**Table 1:** The pH, electrical conductivity (EC), and soil texture class of soil samples collected from Na Si Nuan Secondary Forest, Maha Sarakham, among the three seasons.

Sampling plot	pH (1:1 H_2_O)			EC (1:5) (dSm^−1^)			Soil texture		
	Hot	Rainy	Cool	Hot	Rainy	Cool	Hot	Rainy	Cool
1	4.8	4.7	5.9	0.12	0.08	0.04	Sandy loam	Loamy sand	Sandy loam
2	5.4	5.5	6.3	0.11	0.10	0.05	Sandy loam	Loamy sand	Loamy sand
3	4.1	4.7	5	0.09	0.02	0.02	Loamy sand	Loamy sand	Sandy loam
4	5.7	4.1	4.9	0.04	0.08	0.01	Loamy sand	Sandy loam	Sandy loam
5	4.4	4.0	4.6	0.02	0.06	0.02	Sandy loam	Loamy sand	Loamy sand
6	4.2	3.8	4.9	0.06	0.07	0.01	Loamy sand	Loamy sand	Sandy loam
7	3.9	3.9	4.6	0.07	0.10	0.03	Loamy sand	Loamy sand	Sandy loam
8	4.0	3.8	4.9	0.08	0.08	0.02	Sandy loam	Loamy sand	Loamy sand
9	3.9	4.1	4.8	0.06	0.06	0.02	Sandy loam	Loamy sand	Sandy loam
10	4.1	3.8	5.0	0.08	0.08	0.02	Sandy loam	Sandy loam	Sandy loam
11	4.2	4.1	4.8	0.06	0.09	0.04	Loamy sand	Sandy loam	Sandy loam
12	4.2	4.0	5.0	0.13	0.08	0.01	Sandy loam	Sandy loam	Loamy sand
13	4.3	4.1	5.4	0.07	0.06	0.02	Sandy loam	Loamy sand	Loamy sand
14	4.3	4.2	6.0	0.08	0.09	0.04	Loamy sand	Loamy sand	Loamy sand
Max.	5.7	5.5	6.3	0.13	0.10	0.05			
Min.	3.9	3.8	4.6	0.02	0.02	0.01			
Ave.	4.4	4.2	5.2	0.08	0.08	0.03

**Table 2:** The organic matter, total nitrogen, available phosphorus, and available potassium of soil samples collected from Na Si Nuan Secondary Forest, Maha Sarakham, among the three seasons.

Sampling Plot	Organic matter (%)			Total nitrogen (%)			Available phosphorus (mgkg^−1^)			Available potassium (mgkg^−1^)		
	Hot	Rainy	Cool	Hot	Rainy	Cool	Hot	Rainy	Cool	Hot	Rainy	Cool
1	1.18	0.80	1.77	0.059	0.040	0.088	7.20	4.30	8.20	44.0	32.0	50.0
2	1.30	1.02	1.30	0.065	0.051	0.065	7.15	4.70	8.40	74.0	48.0	86.0
3	0.75	0.57	0.77	0.037	0.028	0.039	9.60	7.05	12.15	45.0	39.0	52.0
4	1.18	0.41	0.88	0.059	0.024	0.044	6.95	3.15	4.50	101.0	35.0	77.0
5	0.73	0.53	0.87	0.036	0.026	0.044	3.30	2.20	3.15	46.0	19.0	28.0
6	0.81	0.62	0.94	0.041	0.031	0.047	3.60	2.00	3.80	21.0	25.0	31.0
7	0.88	0.67	1.04	0.044	0.034	0.052	3.25	2.45	4.30	50.0	35.0	43.0
8	0.90	0.47	0.83	0.045	0.024	0.042	4.95	2.60	3.15	40.0	19.0	29.0
9	0.75	0.50	0.90	0.038	0.025	0.045	3.10	2.20	2.40	44.0	38.0	45.0
10	0.73	0.58	1.41	0.036	0.029	0.070	3.65	2.90	3.15	41.0	36.0	48.0
11	0.84	0.86	1.27	0.042	0.043	0.063	3.65	2.35	2.50	40.0	40.0	52.0
12	1.04	0.70	0.91	0.052	0.035	0.040	4.35	1.85	3.40	78.0	28.0	45.0
13	0.98	0.68	1.00	0.049	0.033	0.043	3.65	2.80	2.45	33.0	21.0	29.0
14	0.83	0.77	0.91	0.041	0.038	0.045	4.25	3.35	3.85	59.0	41.0	48.0
Max.	1.30	1.02	1.77	0.065	0.051	0.088	9.60	7.05	12.15	101.0	48.0	86.0
Min.	0.73	0.41	0.77	0.036	0.024	0.039	3.10	1.85	2.40	21.0	19.0	28.0
Ave.	0.92	0.66	1.06	0.046	0.033	0.052	4.90	3.14	4.67	51.1	32.6	47.4

**Table 3:** An interpretation of physical and chemical properties of soil samples. Range indicates the lowest and the highest values of each parameter within 14 sampling sites.

Parameter	Range	Average/Interpretation
pH	4.2–5.2	4.6/Low^a^
Electrical conductivity (dSm^−1^)	0.03–0.08	0.06/Non-saline^b^
Soil texture class	Loamy sand - sandy loam	-/Coarse^c^
Organic matter (%)	0.66–1.06	0.88/Low^a^
Total nitrogen (%)	0.033–0.052	0.044/Low^a^
Available phosphorus (mgkg^−1^)	3.14–4.90	4.24/Low^a^
Available potassium (mgkg^−1^)	32.6–51.1	43.7/Low^a^

a Interpretation of soil properties from Land Classification Division and FAO Project Staff [1].

b Interpretation of soil properties from Landon [2].

c Interpretation of soil properties from Soil Survey Division Staff [3].

The density of non-, slightly-, and moderately halophilic bacteria of soil collected in three seasons from the study site are summarized in Table 4. The fifty six bacterial isolates were obtained through bacterial enumeration processes. From an initial observation of the Gram stain status and morphological features of the bacterial isolates using a light microscope, most of them were Gram-positive endospore-forming rods. A one-way ANOVA was used to analyze the difference of soil properties and halophilic bacterial density among the three seasons (Table 5).

**Table 4:** Seasonal dynamics of non-, slightly-, and moderately halophilic bacterial density of soil samples collected from Na Si Nuan Secondary Forest, Maha Sarakham, among the three seasons. Values are reported as log CFU g^−1^ of dry soil.

Sampling plot	Non halophilic bacteria			Slightly halophilic bacteria			Moderately halophilic bacteria		
	Hot	Rainy	Cool	Hot	Rainy	Cool	Hot	Rainy	Cool
1	6.72	5.70	6.16	4.91	3.94	4.44	3.97	3.23	3.69
2	5.78	5.32	5.91	4.81	4.15	4.59	3.94	3.11	3.72
3	6.78	5.53	5.53	4.66	3.96	4.26	3.96	3.36	3.76
4	6.70	5.30	6.44	4.21	3.26	3.85	3.89	3.26	3.56
5	5.98	5.28	5.74	4.28	3.18	3.71	3.63	3.40	3.64
6	5.54	5.15	5.35	3.95	3.33	3.63	3.60	3.38	3.53
7	6.45	6.02	6.22	4.25	3.27	3.97	3.78	3.52	3.52
8	6.83	6.19	6.75	4.51	3.11	3.91	3.89	3.57	3.57
9	6.88	5.71	6.65	4.08	3.38	3.69	3.50	3.30	3.30
10	5.92	5.50	5.70	5.52	5.09	5.52	3.82	3.18	3.75
11	6.87	5.83	6.82	4.58	3.98	4.45	3.83	3.39	3.39
12	7.04	5.75	6.47	4.27	3.86	4.29	3.88	3.29	3.72
13	6.45	5.66	6.20	4.83	4.14	4.53	3.85	3.20	3.50
14	6.26	5.28	6.18	4.80	4.03	4.66	3.85	3.23	3.73
Max.	7.04	6.19	6.82	5.52	5.09	5.52	3.97	3.57	3.76
Min.	5.54	5.15	5.35	3.95	3.11	3.63	3.50	3.11	3.30
Ave.	6.44	5.59	6.15	4.55	3.76	4.25	3.81	3.32	3.60

**Table 5:** Statistical analysis of soil properties and halophilic bacterial density among the three seasons.

Parameters	Hot	Rainy	Cool
pH	4.4^b^	4.2^b^	5.2^a^
Electrical conductivity (dSm^−1^)	0.08^a^	0.08^a^	0.03^b^
Organic matter (%)	0.92^a^	0.66^b^	1.06^a^
Total nitrogen (%)	0.46^a^	0.033^b^	0.052^a^
Available phosphorus (mgkg^−1^)	4.99^a^	3.14^a^	4.67^a^
Available potassium (mgkg^−1^)	51.1^a^	32.6^b^	47.4^ab^
Non-halophilic bacteria (log CFUg^−1^ dry soil)	6.44^a^	5.59^b^	6.15^a^
Slightly-halophilic bacteria (log CFUg^−1^ dry soil)	4.55^a^	3.76^b^	4.25^a^
Moderately-halophilic bacteria (log CFUg^−1^ dry soil)	3.81^a^	3.32^c^	3.60^b^

^abc^ Values with the same letter within rows indicate no significant difference with *P* ≥ 0.05.

## 2 Experimental design, materials and methods

### 2.1 Description of sampling area

**Fig. 1:**
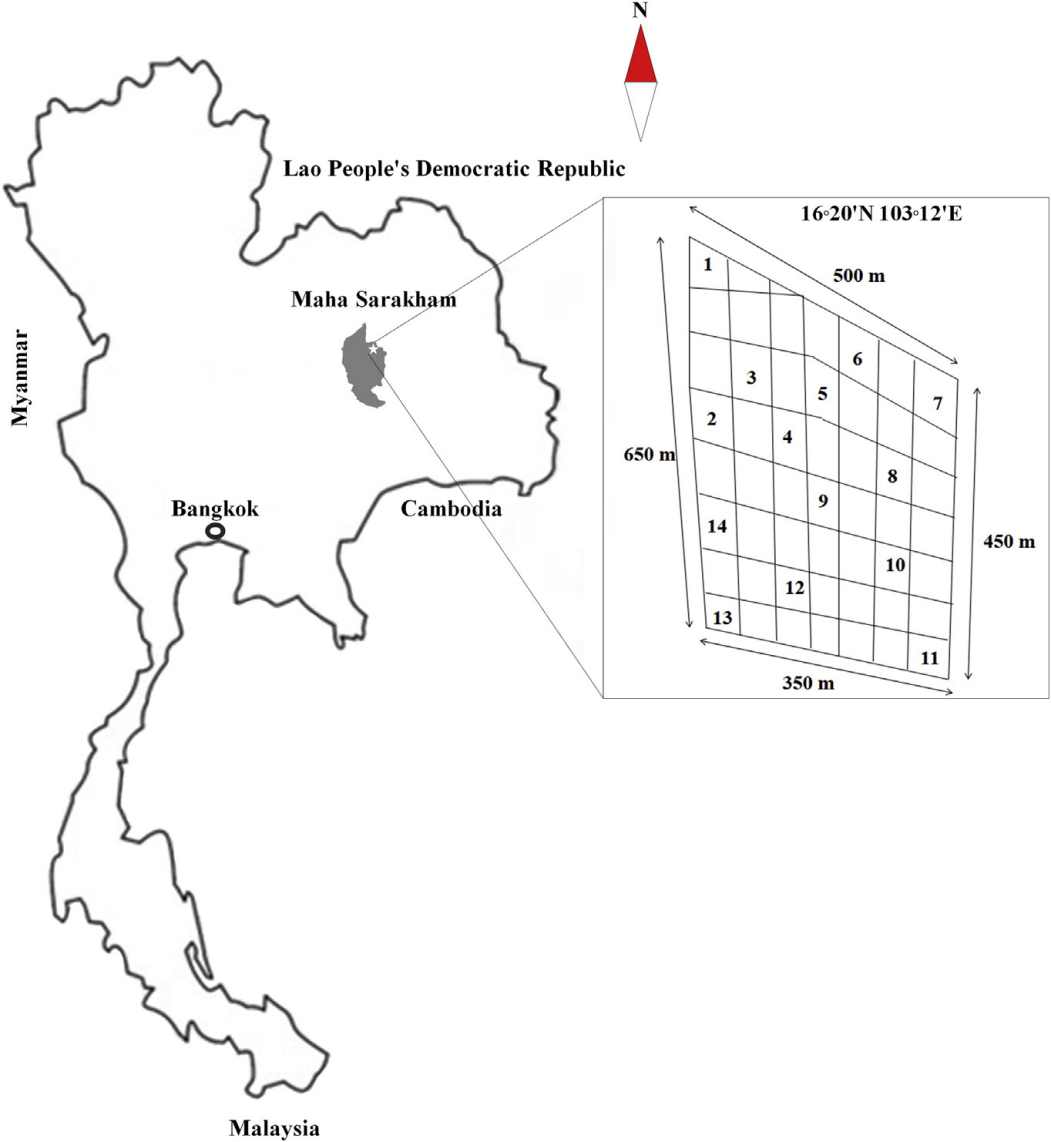
Map of the study area: shaded area depicts the location of Na Si Nuan Secondary Forest at Maha Sarakham Province, Thailand.

The sampling area, Na Si Nuan Secondary Forest at Maha Sarakham Province, Thailand, is situated between latitude 16°20′N and longitude 103°12′E with a total area of approximately 19.2 ha. The sampling sites were divided into 14 plots (Fig. 1).

### 2.2 Sample collection and analytical procedures

Soil samples were collected seasonally during June 2017 to March 2018. The samples were randomly taken from three subplots for each sampling site at a depth of 30 cm using a hand auger. Samples from the same sampling site were mixed thoroughly to obtain the composite sample. Then a portion (one kilogram) of the composite soil samples was collected and stored at 4 °C for further examination.

#### 2.2.1 Physical and chemical analysis of soil samples

The pH, electrical conductivity (EC), soil texture class, organic matter, total nitrogen, available phosphorus and available potassium of the soil samples were determined seasonally. Details of the methods of physical and chemical analysis are given elsewhere on Page et al. [4,5] and Division of Soil analysis [6].

#### 2.2.2 Density of culturable halophilic bacteria

The number of non-, slightly-, and moderately halophilic bacteria in soil samples were enumerated using a spread plate technique. The halobacteria medium [7] containing NaCl at 0, 3, and 6% (wv^−1^) were used for enumeration of non-, slightly-, and moderately halophilic bacteria, respectively. The NaCl concentrations used were chosen from the level of salt requirements for the growth of each group of halophilic bacteria [8]. After incubation at 37 °C for 2–3 days, the colony forming units (CFU) were counted. Then the density of culturable halophilic bacteria were calculated and reported as log CFUg^−1^ of dry soil. Different colonies grown on media were selected and purified for further characterization.

#### 2.2.3 Data analysis

The values of soil physical and chemical properties and density of halophilic bacteria of each plot were averaged from its subplots. Data obtained from three seasons were compared by one-way analysis of variance (ANOVA) and the significance of mean difference among the three seasons was done by multiple comparison tests (Tukey's HSD Post Hoc Test). Statistics analyses were performed using the SPSS version 17.0 (SPSS Inc., USA.).
